# Metastasis to the pancreas: a rare site for secondary malignancy of breast cancer (a case report)

**DOI:** 10.11604/pamj.2020.37.260.25228

**Published:** 2020-11-23

**Authors:** Fadila Kouhen, Meriem Chihabeddine, Mohammed Squali, Mohammed Allaoui, Abderrahmane Al Bouzidi, Nadia Errafiy, Nabil Ismaili

**Affiliations:** 1 Mohammed VI University of Health Sciences (UM6SS), Department of Radiotherapy, International University Hospital Sheikh Khalifa, Casablanca, Morocco,; 2 Mohammed VI University of Health Sciences (UM6SS), Department of Medical Oncology, International University Hospital Sheikh Khalifa, Casablanca, Morocco,; 3 Faculty of Medicine and Pharmacy, Mohammed V University, Rabat, Morocco,; 4 Department of Pathology, Military Hospital Mohammed V, Rabat, Morocco,; 5 Mohammed VI University of Health Sciences (UM6SS), National Reference Laboratory (LNR), Casablanca, Morocco,

**Keywords:** Breast cancer, pancreatic metastases, prognosis, case report

## Abstract

Breast cancer is the most frequent invasive cancer in women and the second cause of death by cancer in women after lung cancer. It causes metastases especially to bones, liver and lungs. Pancreatic metastases from a primary breast neoplasm are rare and unusual, occurring in less than 3% of the cases. There have been only 28 cases described in the literature. This paper adds one more case to the published literature. We present a case of pancreatic metastasis of the breast in a 64-year-old female and a discussion based on a review of the literature.

## Introduction

With two million new cases in 2018, breast cancer is the most frequent invasive cancer in women and the second cause of death by cancer in women after lung cancer [1]. The screening and the treatment improvement decreased the breast cancer mortality, however 20 to 30% of patients develop a distant metastasis [1]. The most common breast cancer metastasis sites are the bones, the lungs, the brain, and the liver. Pancreatic metastases from a primary breast cancer are rare, occurring in less than 3% of the cases [2]. There have been only 28 cases described in the literature. This paper adds one more case to the published literature. We present a case of pancreatic metastasis of the breast in a 64-year-old female and a discussion based on a review of the literature.

## Patient and observation

A 64-year-old post-menopausal female patient, with no significant past medical or family history was admitted to our hospital with the following medical history. Her history dates back 1 year when she detected a lump in her left breast. Five months after, she reported severe lower back pain with jaundice, nausea and loss of nine kilograms in four months. The physical examination showed a retro-nipple mass of 5 cm in diameter in the left breast with frank cutaneous-mucosal jaundice and the abdomen was painful to deep palpation. Neurologic examination revealed no focal deficits. Mammography analysis revealed a retro-nipple lesion of the left breast classified ACR 5. After fine-needle aspiration, invasive lobular breast carcinoma was detected in pathological examination. Immunohistological staining revealed that hormone receptors were positive, with estrogen receptors (ERs) at: 80% and progesterone receptors (PgRs) at 30%. The HER2 score was 1+ and the Ki67 was at 22%. Abdominal computed tomography demonstrated a hyper vascularized, irregular solid lesion of 4.3 cm x 2.7 cm x 7.0 cm in the head of the pancreas with discreet biliary duct dilation. In addition, Bones scintigraphy showed abnormal increased accumulation of radiopharmaceutical along the right humeral head, L4, and femurs

**Figure 1:**
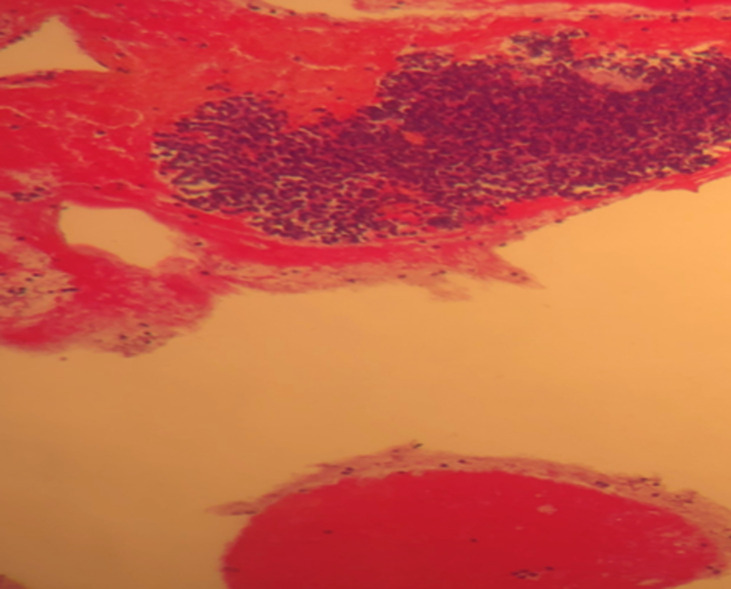
infiltration by a tumour composed of nests and foci of cells with hyperchromatic nuclei (HE, Gx40)

**Figure 2:**
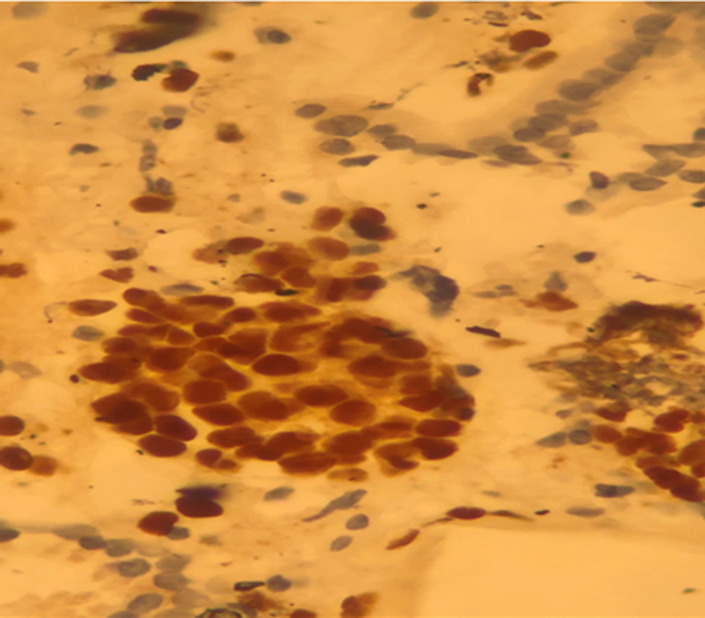
Ki-67 labelling index is high

**Figure 3:**
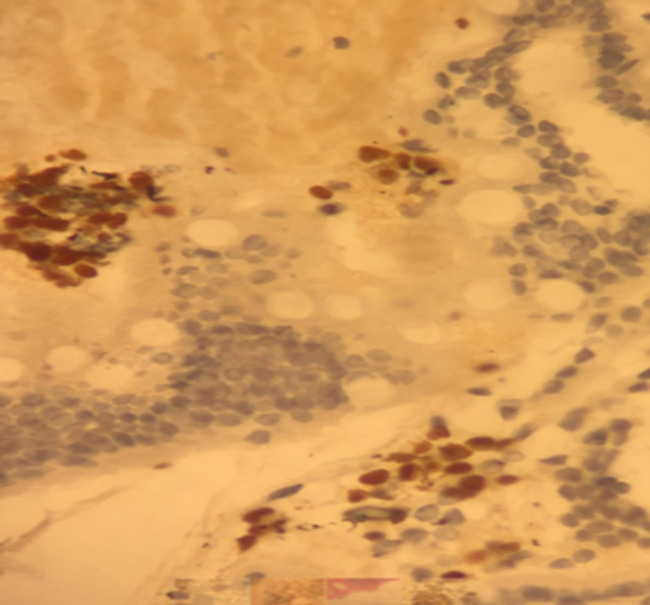
estrogen receptor expression by the tumor cells

Lumbar spine MRI evaluation showed a tumor process involving the vertebral body and the posterior arch of L4. Histopathology by pancreatic endoscopic ultrasound-guided fine needle biopsy confirmed metastatic carcinoma with breast origin. The morphological and immunohistochemical features of pancreatic-metastasis were similar to the primary carcinoma breast (Figure 1, Figure 2, Figure 3). Cancer antigen (CA)-19.9, carcinoembryonic antigen (CEA) and CA-15-3 levels were in the normal range. She underwent percutaneous transhepatic biliary drainage and fluoroscopic guided stenting with a metallic-stent (ELLA stent). The liver-functions normalized gradually and patient´s general-condition also improved.

Our Therapeutic management was started firstly by the placement of a biliary prosthesis, then by analgesic and decompressive conventional radiotherapy at L4 with radiation dose of 30 Gy (3 Gy/fraction). A systemic therapy consisting of letrozole 2.5 mg/day combined with palbociclib 25 mg/day (3 weeks ON and one week OFF) and denosumab at a dose of 120 mg/month was prescribed. The treatment was well tolerated except grade 1 anemia and fatigue. The first assessment three months after starting treatment showed a partial response according to the response evaluation criteria in solid tumors (RECIST) V1.1 criteria. After 22 months of follow-up, the evolution was marked by lesion stability without local and distant disease progression.

## Discussion

Pancreatic metastases from other primary malignancies are uncommon and they don´t exceed 2% of pancreatic cancers [2]. The most common primary cancers with pancreatic metastases are kidney cancer, followed by colorectal cancer, melanoma, breast cancer, lung carcinoma and sarcoma [3]. Breast cancer causes metastases especially to bones, liver and lungs. Pancreatic metastases from a primary breast neoplasm are rare and unusual, occurring in less than 3% of the cases. To our knowledge, 29 cases of pancreatic metastases from breast cancer, including the present case, have been reported at 17 case reports and 6 case series since the first report in 1982 by Azzarelli *et al*. [4]. Typically, invasive lobular breast carcinoma is the most common type of breast cancer metastasizes to the pancreatic gland [5]. The clinical presentation is similar for both primary and secondary neoplasms. Most patients present with obstructive jaundice caused by compression of the bile duct in the head of the pancreas. The patient can have also epigastric or back pain, and weight loss [6].

In recent years, diagnostic imaging techniques such as Doppler ultrasound (US), helical computed tomography (CT), enhanced magnetic resonance imaging (MRI), and endoscopic US (EUS) have been developed, elevating the ability to diagnose pancreatic tumors, but it is often difficult to distinguish pancreatic metastasis from a primary pancreatic tumor [7,8]. Our patient had undergone an endoscopic ultrasound with fine needle aspiration which had confirmed a diagnosis of pancreatic metastases of breast cancer. Surgical treatment is indicated when the pancreatic lesion is single and for patients fit to perform a pancreatectomy [9,10]. For our patient, surgery was not indicated due to several metastatic sites.

The prognosis for patients with pancreatic metastatic disease is usually better than for patients with primary pancreatic tumors. Masetti *et al*. analyzed the prognostic factors relating to metastatic tumors to the pancreas, and found at univariate survival analysis a 2-years probability of survival of 57.1% in pancreas metastases from breast cancer and a 5-years probability of survival of 34.3% [11]. Palbociclib, highly selective inhibitors of CDK4 and CDK6, serine-threonine kinases that regulate the cell cycle progression, have been approved in recent years for the treatment of endocrine-resistant MBC in combination with endocrine therapy considering their efficacy in prolonging progression-free survival, increasing clinical benefit rate and response rate in different clinical context and treatment lines [12-14]. Our patient remained without disease progression at the time of her last follow-up examination (22 months after diagnosis).

## Conclusion

In summary, this case demonstrates the very rare case of a breast cancer metastasis to the pancreas arising as the first symptom of metastatic breast cancer. Pancreatic metastasis is difficult to diagnose, because its clinical and radiological presentation is similar to that of a primary pancreatic tumor. Thus, the clinician should suspect the possibility of pancreatic metastasis in cases of pancreatic lesions detected in any patient with a prior history of cancer, including breast cancer.
